# Efficacy of generic sofosbuvir with daclatasvir compared to sofosbuvir/ledipasvir in genotype 4 hepatitis C virus: A prospective comparison with historical control

**DOI:** 10.1002/hsr2.980

**Published:** 2022-12-08

**Authors:** Hala Joharji, Delal Alkortas, Aziza Ajlan, Mohamed Ahmed, Hamad Al‐Ashgar, Mohammed Al‐Quaiz, Dieter Broering, Mohammed Al‐Sebayel, Hussien Elsiesy, Faisal A. Alkhail, Waleed K. Al‐Hamoudi, Edward De Vol, Epi Amirah Almuhayshir, Ahmed Al‐Jedai

**Affiliations:** ^1^ King Faisal Specialist Hospital and Research Centre Organ Transplant Center of Excellence Riyadh Saudi Arabia; ^2^ Department of Medicine King Faisal Specialist Hospital and Research Centre Riyadh Saudi Arabia; ^3^ Liver and Small Bowel Transplant and Hepatology Surgical Department King Faisal Specialist Hospital and Research Centre Riyadh Saudi Arabia; ^4^ Biostatistics, Epidemiology and Science Computing Department King Faisal Specialist Hospital and Research Centre Riyadh Saudi Arabia; ^5^ Therapeutic Affairs, Ministry of Health Riyadh Saudi Arabia; ^6^ Alfaisal University Colleges of Medicine and Pharmacy Riyadh Saudi Arabia

**Keywords:** cost saving, direct acting antivirals, sustained virological response, viral hepatitis

## Abstract

**Background and Aim:**

Management of genotype 4 hepatitis C virus (HCV) has shifted to interferon‐free regimens with a high sustained virological response (SVR‐12), especially with NS5B/NS5A inhibitor combinations such as sofosbuvir and ledipasvir (Sof‐Led). The guidelines have recommended the combination of sofosbuvir and another NS5A inhibitor, daclatasvir, to manage HCV genotypes 1–3. However, its use was extended to genotype 4 HCV based on extrapolating evidence. Our aim is to assess the efficacy of generic sofosbuvir + branded daclatasvir (Sof‐Dac) compared to the Sof‐Led combination in treating genotype 4 HCV.

**Methods:**

This study is an open‐label, 2‐period, noninferiority study that compared patients receiving a combination of generic sofosbuvir 400 mg and daclatasvir 60 mg orally daily (Group 2) prospectively to a historical control (Group 1) that included patients who received a combination of sofosbuvir/ledipasvir 400/90 mg orally daily. The primary endpoint is the proportion of patients who achieved SVR‐12.

**Results:**

The study included 111 patients in the (Sof‐Led) Group 1 and 109 patients (Sof‐Dac) Group 2. For the primary outcome, SVR‐12 was achieved in 106 (95.5%) of the patients in Group 1 versus 108 (99.1%) in Group 2 (*p* = 0.2). In addition, all patients who achieved SVR‐12 also achieved SVR‐24.

**Conclusion:**

Generic sofosbuvir combined with branded daclatasvir was safe and effective for treating genotype 4 HCV compared to Sof‐Led. This combination may significantly reduce the cost burden, enabling a larger pool of treated patients. Office of research affairs at KFSHRC RAC# 2171 036.

## 1 INTRODUCTION

Chronic hepatitis C virus (HCV) infection affects 58 million people universally; it is associated with a high mortality rate reaching 290,000 deaths annually.^1^ In patients with long‐standing, well‐established HCV infection, disease progression is associated with considerable morbidity and a high‐cost burden.^2^ HCV complicated by cirrhosis or hepatocellular carcinoma is one of the most common liver transplantation indications.^3^, ^4^ The risk of recurrence of HCV posttransplant is considerably high in all patients; however, the risk is even higher in those patients who were viremic at the time of transplantation, leading to a worse prognosis of the disease and graft loss.^5^

There have been multiple studies that aimed to determine the prevalence of HCV in Saudi Arabia. The overall prevalence is estimated to fall between 0.3% and 1.1%.^6^, ^7^, ^8^ HCV genotype 4 was identified as the most prevalent genotype in the Saudi population, followed by genotype 1. Genotypes 2a/2b emerged from the Eastern Province and genotype 5 from the Western Province, whereas genotypes 3 and 6 remain extremely rare.^9^, ^10^, ^11^, ^12^

The treatment of HCV has rapidly evolved over the past several years. For decades, treatment options were limited to ribavirin/interferon combination, which was poorly tolerated and required prolonged therapy (up to 48 weeks for genotypes 1 and 4) with a reduced response rate; sustained virological response (SVR) of less than 50%. The approval of new oral, direct‐acting antiviral (DAA) agents has revolutionized patients' management of HCV. DAAs are associated with better safety and efficacy profiles, shorter treatment duration, and improved outcomes (SVR > 90%).^13^, ^14^, ^15^, ^16^

Patients with HCV genotype 4, the most predominant genotype in Saudi Arabia, are poorly represented in most clinical trials involving DAAs. The American Association for the Study of Liver Disease includes sofosbuvir/ledipasvir (Sof‐Led) as one of the first‐line therapies for managing HCV genotype 4, but not sofosbuvir and daclatasvir combinations.^17^ Despite the revolutionary nature of DAA therapy, one of the main challenges regarding adopting DAAs has been the high cost of these medications. In 2016, generic sofosbuvir became available in Saudi Arabia, significantly reducing HCV treatment's overall cost. Our institution has adopted generic sofosbuvir plus daclatasvir as the first‐line regimen to treat HCV genotype 4. Despite the favorable cost savings potential, there were some concerns about the efficacy of the generic sofosbuvir and daclatasvir combination compared to the previous first‐line regimen in our institution of sofosbuvir/ledipasvir.

This study's main objective was to assess generic sofosbuvir's efficacy and safety plus daclatasvir (Sof‐Dac) compared with (Sof‐Led).

## 2 METHODS

### 2.1 Design and settings

This study is an open‐label, 2‐period, noninferiority study that included all patients who received a combination of sofosbuvir/ledipasvir 400/90 mg orally daily (Group 1) from January 1st, 2015 to September 30th, 2016 (historical control arm) to patients receiving a combination of sofosbuvir 400 mg and daclatasvir 60 mg orally daily (Group 2, prospective arm) starting from October 1st, 2016 until March 31st, 2018 at King Faisal Specialist Hospital and Research Center, a tertiary care hospital in Riyadh, Saudi Arabia.

### 2.2 Study population

All adult patients >18 years old who were followed at KFSHRC for HCV treatment with or without cirrhosis were included regardless of the treatment history. In addition, postliver transplant patients were included if HCV was the main indication for transplantation. Pediatric patients, patients coinfected with human immunodeficiency virus or hepatitis B virus, patients with previous exposure to an NS5A inhibitor, patients with decompensated cirrhosis, patients with glomerular filtration rate eGFR <30 ml/min/1.73 m^2^, and nongenotype 4 HCV patients were excluded.

### 2.3 Outcomes

The primary study endpoint was the proportion of patients achieving an SVR‐12 defined as serum HCV RNA below the lower limit of quantification (LLoQ, 30 IU/ml) 12 weeks after the end of 12 weeks of therapy (EOT). Secondary endpoints were the proportion of patients achieving an SVR‐24 defined as serum HCV RNA below the lower limit of quantification (LLoQ, 30 IU/ml) 24 weeks after the end of therapy, the change in Child Turcotte Pugh (CTP) scores at Week 12 after the end of the treatment period in cirrhotic patients, documented adverse events until Day 30 after the end of treatment with both regimens, and serum HCV RNA levels measured by a real‐time polymerase chain reaction test. The SVR is defined as achieving aviremia and viral load detection 12 weeks after EOT. Failure to achieve SVR could reflect relapse, recurrence, or nonresponse. Relapse is defined as achieving early virological response (EVR) at 1 month (4 weeks after initiation of treatment), but EOT response. Recurrence is defined as failure to achieve SVR‐12 after a prior achievement of EVR and EOT response. Nonresponse is defined as failure to achieve both EVR and EOT response. Any adverse event was systematically captured for all patients during clinic visits in the prospective group. For the historical group, we depended on documentation in the electronic patient record.

### 2.4 Statistical analysis and data collection

The study was designed to include 102 subjects in each group. The sample size was determined based on a noninferiority margin of 9% between the proportions of SVR‐12 in the two treatment groups and type I and type II errors rates of 0.05 and 0.2, respectively. Unless noted otherwise, a comparison of continuously‐scaled data between the two treatment groups was described with means and standard deviations and evaluated with two‐sample *t*‐tests. Inferential methods were nonparametric‐based, that is, baseline comparison of continuously‐defined data was measured with Wilcoxon's Rank‐Sum test. Baseline comparisons of categorical parameters were measured with percentages evaluated with Fisher's Exact Test. The differences in the primary and secondary endpoints were evaluated with Fisher's Exact Test. The calculated *p*‐values are presented without adjustment for multiple comparisons for all tests. A decision of statistical significance was based on *p*‐values no larger than 0.01 to account for the multiple testing.

Included patients in the historical control group were identified through Electronic Health Records. While eligible patients for treatment with Sof‐Dac prospectively were identified through the HCV clinic visits. Data were collected using a standardized electronic data collection form through an integrated clinical information system (ICIS), electronic medication administration records (eMAR), and chart review. Data were analyzed utilizing SPSS software.

### 2.5 Ethical considerations and patient confidentiality

This study was approved by the institutional review board of King Faisal Specialist Hospital and Research Centre (KFSHRC) and conducted following the latest version of the Declaration of Helsinki and Good Clinical Practice^18^ and the policies and procedures of the Office of Research Affairs at the KFSHRC, Riyadh, Saudi Arabia, and the laws and regulations of Saudi Arabia. RAC# 2171 036. All authors had access to the study data and reviewed and approved the final manuscript. Informed consent was waived since the prospective arm was noninterventional, and enrolled patients received the standard of care according to our institutional guidelines.

## 3 RESULTS

**Figure 1:**
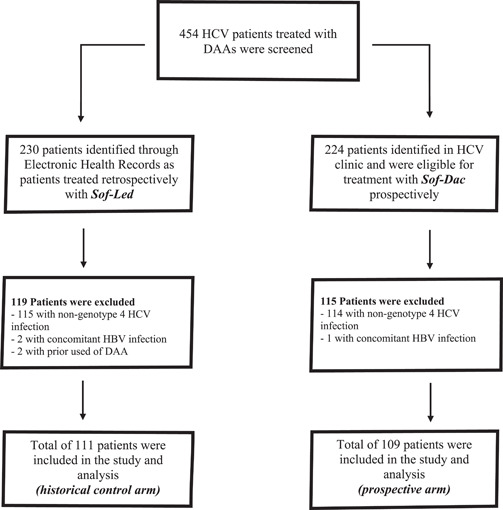
Schematic diagram of patient's enrolment

During the study period, we included 111 patients in Group 1 and 109 patients in Group 2 (Figure 1). Baseline demographics are presented in Table 1.

**Table 1:** Baseline characteristics—all included patients

	Sof‐Led *n* = 111	Sof‐Dac *n* = 109	*p*‐Value
**Age (years), median and interquartile range**	61.9 (55.5–67.7)	56.1 (36.5–65.6)	0.008
**Gender**			
Male, *n* (%)	37 (33.3%)	50 (45.9%)	
Female *n* (%)	74 (66.7%)	59 (54.1%)	0.07
**Body mass index (m^2^), mean**	29.0 ± 6.5	28 ± 7.7	0.2
**HCV RNA at baseline (IU/ml), mean**	2,224,589 ± 3,420,526	2,123,646 ± 6,641,342	0.8
**Past treatment status (%)**			
Treatment naïve	83 (74.8%)	92 (84.4%)	0.09
Peg‐interferon‐based regimens experienced	28 (25.2%)	17 (16.6%)	
**Experienced outcome (%)**			
Relapse	11 (39.3%)	5 (29.4%)	0.5
Null response	10 (35.7%)	4 (23.5%)	0.5
Partial response	5 (17.9%)	5 (29.4%)	0.4
Intolerance	2 (7.1)	2 (11.8%)	>0.99
Undetermined	0	1 (5.9%)	
			Overall *p*‐value 0.5
**Used with ribavirin (%)**	69 (62.2%)	38 (34.9%)	<0.001
**Duration of therapy (%)**			
8 weeks	6 (5.4%)	0	0.02
12 weeks	102 (91.9%)	108 (99.1%)	0.01
12 weeks ***(repeated course)***	3 (2.7%)	1 (0.9%)	0.6
			Overall *p*‐value 0.2
**Treated for HCV postliver transplant (%)**	22 (19.8%)	6 (5.5%)	0.002
**MELD score (mean)**	9.47 ± 4.23	8.50 ± 3.74	0.07
**Labs and biomarkers (mean)**			
Alanine aminotransferase (ALT) (IU/L)	58.4 ± 61.9	44.4 ± 39.9	0.04
Aspartate aminotransferase (AST) (IU/L)	60.6 ± 52.8	49.3 ± 42.5	0.08
Alkaline phosphatase (IU/L)	126 ± 64	115 ± 84	0.2
Albumin (g/L)	36.8 ± 6.3	39.6 ± 5.8	0.001
Total bilirubin (mg/dl)	20.8 ± 22.6	16.8 ± 23.9	0.1
Hemoglobin (g/dl)	129 ± 23	130 ± 22	0.6
International normalized ration (INR)	1.2 ± 0.2	1.1 ± 0.2	0.05
Serum creatinine (umol/l)	74.7 ± 23.6	71.8 ± 22.4	0.3
**CTP class, *n* (%)**			
A (5–6)	37 (60.7%)	21 (58.3%)	0.1
B (7–9)	23 (37.7%)	15 (41.7%)	0.1
C (10–15)	1 (1.6%)	0	>0.99
			Overall *p*‐value 0.1
**Estimated METAVIR fibrosis score, *n* (%)** a			
F0	12 (12.8%)	24 (24.7%)	0.04
F1	7 (7.4%)	23 (23.7%)	0.002
F2	14 (14.9%)	14 (14.4%)	>0.99
F3	20 (21.3%)	7 (7.2%)	0.006
F4	41 (43.6%)	29 (29.9%)	0.05
			Overall *p*‐value < 0.001

Abbreviations: AIH, autoimmune hepatitis; CTP, Child‐Turcotte‐Pugh Score; HCC, hepatocellular carcinoma; HCV, hepatitis C virus; MELD, model for end‐stage liver disease; Sof‐Dac, sofosbuvir (Sovira®) + daclatasvir (Daclinza®); Sof‐Led, sofosbuvir/ledipasvir (Harvoni®).

^a^ Using transient elastography (Fibroscan Echosens).

The median (and interquartile range) for age in Group 1 was 61.9 (55.5–67.7) compared to 56.1 (36.5–65.6) in Group 2 (*p* = 0·008). Females were the majority in both treatment groups—Group 1, numbering 74 (66.7%), and Group 2 with 59 (54.1%) (*p* = 0.07). The mean baseline HCV RNA viral load was (6.3 log IU/ml ± 6.5 log IU/ml) in Group 1 versus (6.3 log IU/ml ± 6.8 log IU/ml) in Group 2 (*p* = 0.8). The majority of patients were treatment naïve in both groups, 83 (74·8%) in Group 1 versus 92 (84·4%) in Group 2 (*p* = 0.09). Ribavirin was used in 69 (62.2%) in Group 1 versus 38 (34.9%) in Group 2 (*p* = 0.0001). The duration of therapy was for 12 weeks in 102 (91.9%) in Group 1 versus 108 (99.1%) in Group 2 (*p* = 0.01). All patients who were treated for 8 weeks in Group 1 achieved SVR‐12. Three patients received a repeated course of Sof‐Led for 12 weeks in Group 1 due to relapse in one patient and no response in two patients. The other six patients were treated for 8 weeks because they were treatment‐naive with low viral load according to the guidelines. In the Sof‐Dac arm, one patient did not achieve SVR in the first 12 weeks due to nonadherence and received a second course of Sof‐Dac for 12 weeks. The SVR‐12 in some baseline populations that were imbalanced at baseline was reported. It was found that 92% of ribavirin users achieved SVR‐12 in the Sof‐Led group versus 100% in Sof‐Dac group. While it was found that 75% achieved SVR‐12 in the Sof‐Led group with a Metavir score of F0 versus 100% in the Sof‐Dac group. For patients with a Metavir score of F1, 100% of the patients achieved SVR‐12 in the Sof‐Led group versus 95% in the Sof‐Dac group. All patients with other degrees of fibrosis, F2, F3, and F4, achieved SVR‐12 in both groups.

A small number of patients have been treated postliver transplantation in both groups: 22 (19.8%) in Group 1 versus 6 (5.5%) in Group 2 (*p* = 0.002). The baseline characteristics of those patients are detailed in Table 2.

**Table 2:** Baseline characteristics for posttransplant treated patients

	Group 1	Group 2	*p*‐Value
	Sof‐Led	Sof‐Dac	
	***n*** = **22**	***n*** = **6**	
**Donor type (%)**			
Living	16 (72.7%)	4 (66.7%)	>0.99
Deceased donor	6 (27.3%)	2 (33.3%)	
**Indication of liver transplant (%)**			
HCV	18 (81.8%)	3 (50%)	0.3
HCV‐AIH	1 (4.6%)	0	
HCV‐HCC	3 (13.6%)	3 (50%)	
**Time from most recent transplant (%)**			
1–2	3 (13.6%)	2 (33.3%)	0.003
>2–4	2 (9.1%)	3 (50%)	
4–6	1 (4.6%)	1 (16.7%)	
>6–8	0	0	
>8–12	0	0	
>12–24	0	0	
>24	16 (72.7%)	0	
**Immunosuppressive drug use, *n* (%)**			
Tacrolimus	10 (45.5%)	1 (16.6%)	0.1
Cyclosporine	4 (18.2%)	0	
Tacrolimus + Mycophenolate	1 (4.5%)	1 (16.7%)	
Tacrolimus + Mycophenolate + Prednisone	7 (31.8%)	4 (66.6%)	
**MELD score (mean)**	11.2 ± 5.7	9.8 ± 6.6	0.6

Abbreviations: AIH, autoimmune hepatitis; CTP, Child‐Turcotte‐Pugh Score; HCC, hepatocellular carcinoma; HCV, hepatitis C virus; MELD, model for end‐stage liver disease; Sof‐Dac, Sofosbuvir (Sovira®) + Daclatasvir (Daclinza®); Sof‐Led, Sofosbuvir/Ledipasvir (Harvoni®).

**Figure 2:**
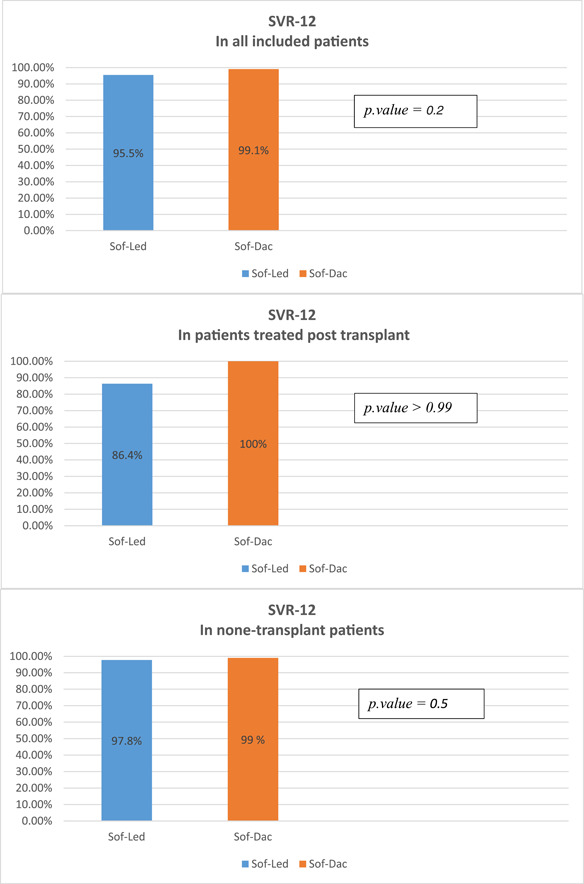
Sustained virological response at 12 weeks after the end of treatment (sustained virological response [SVR‐12]) in sofosbuvir/ledipasvir (Sof‐Led) treated group versus generic sofosbuvir with daclatasvir (Sof‐Dac) treated group

For the primary outcome in all included patients, SVR‐12 was achieved in 95.50% of the patients in Group 1 versus 99.1% in Group 2 (*p* = 0.2) (Table 3; Figure 2). A 95% confidence interval for the difference between the two proportions is (−8.3% and 1.3%). Additional analysis was done for SVR‐12 for patients treated posttransplant and none transplant patients separately. Sustained virological response (SVR‐24) was achieved in all patients who achieved SVR‐12. In Group 1, there was one patient with relapse, two patients with no response throughout the course of treatment, and one who did not have viral load measured at 12 weeks after the end of treatment but only at 24 weeks; while in Group 2, one patient did not achieve SVR‐12 due to nonadherence issues and therefore was considered a nonresponder with treatment failure. None of the patients developed recurrence or relapse as of the last follow‐up. For the secondary endpoint, the effect on CTP scores at the time of SVR‐12: 28 subjects (22 in Group 1 and 6 in Group 2) received liver transplants and were excluded from this analysis. This leaves 41 with a METAVIR score of F4 in Group 1 versus 28 in Group 2. The changes in the CTP at 12 weeks for these two groups were: two improving, three worsening, and 36 no change in Groups 1 and 2, 2 and 24 in Group 2 (*p* > 0.99). In Group 1 (Sof‐Led), two patients had improved CTP scores moving from score B to score A. Both were treatment naïve and received 12 weeks of treatment, and one was treated with ribavirin. On the other hand, three patients had worsened CTP scores: two from score A to B, and one from score B to C. One of those who went from A to B received 8 weeks of treatment. All of them were treatment naïve. In Group 2 (Sof‐Dac), two patients had improved CTP scores; both scores moved from score B to score A. Both patients were treatment naïve and received 12 weeks course of treatment. While two patients had worsened CTP scores: one went from score A to B, and one went from score B to C. These patients were also treatment naïve and received 12 weeks of therapy. No major adverse events were reported in either treatment group. However, lethargy in one patient leading to medication nonadherence, headache in two patients, and pruritus in one patient were reported in Group 2. In contrast, no patient reported any adverse events of these types in Group 1.

**Table 3:** Primary endpoint: Sustained virological response at 12 weeks after the end of treatment (SVR‐12)

	Group 1	Group 2	
	Sof‐Led *n* = 111	Sof‐Dac *n* = 109	*p*‐Value
**SVR‐12 (%)**	106 (95.5%)	108 (99.1%)	0.2
‐Patients treated postliver transplantation *n* = 28	19 (86.4%)	6 (100%)	>0.99
‐None transplant patients *n* = 192	87 (97.8%)	102 (99.1%)	0.5

Abbreviations: Sof‐Dac, Sofosbuvir (Sovira®) + Daclatasvir (Daclinza®); Sof‐Led, Sofosbuvir/Ledipasvir (Harvoni®); SVR‐12, Sustained virological response 12 weeks after the end of treatment.

## 4 DISCUSSION

Hepatitis C infection cure became achievable after the introduction of DAA therapy. Genotype 4 HCV infection is the most prevalent genotype in the Middle East, unlike its prevalence worldwide.^12^ The current American Association for the Study of Liver Diseases and the European Association for the Study of the Liver guidelines recommend certain combinations of (NS5A/NS5B) protein inhibitors as one of the first‐line treatment options for managing genotype 4 HCV infections^19^, ^20^ Although the newer recommendations in AASLD are moving toward utilizing pan‐genotypic co‐formulated combinations, such as glecaprevir‐pibrentasvir and sofosbuvir‐velpatasvir, however, the EASL guidelines recognize that treatment access may be limited in low‐and middle‐income countries. For that, the pan‐genotypic combination of generic sofosbuvir and generic daclatasvir is recommended for use in line with the 2016 EASL recommendations.

Our results indicate that the treatment of genotype 4 HCV infection with the combination of Sof‐Dac is as effective as the standard of therapy. In this study, the combination of generic sofosbuvir and branded daclatasvir (Sof‐Dac) resulted in an SVR‐12 of 99% compared to 96% in the Sof‐Led group.

Multiple studies reported similar SVR‐12 using (NS5A/NS5B) protein inhibitor combinations. SOLAR‐2, an observational study that included 35 patients with genotype 4 HCV infection with advanced liver disease, supports the efficacy of Sof‐Led with ribavirin in treating this population. Patients treated for 12 weeks achieved SVR‐12 in 78% of the cases compared to 94% in patients treated for 24 weeks.^21^ Likewise, results were reported with the Sof‐Led combination in the SYNERGY trial that included genotype 4 HCV infected patients, treatment naïve, and interferon treatment‐experienced patients with an SVR‐12 of 100%.^22^

The IMPACT, CUPILT, and ENDURANCE‐3 studies reported the initial efficacy data on Sof‐Dac in genotypes 1, 2, or 3 with high SVR‐12 proportions in the range of 96%–100% when combined with simeprevir or alone. However, data are still scarce on genotypes 4.^16^, ^23^, ^24^ The combination of Sof‐Dac in treating genotype 4 HCV‐infected patients with compensated cirrhosis for 12 weeks was reported in an observational single‐arm study in 183 patients with an SVR‐12 of 96%, excluding patients with advanced liver disease.^25^

Our study elected to use the generic sofosbuvir and branded daclatasvir as a possible suitable NS5B/NS5A alternative for cost minimization and noninferior outcomes. Some recently published reports assessed the efficacy of a generic combination of DAA. Three Egyptian retrospective studies evaluated the effect of using generic DAA in genotype 4 HCV. First was a retrospective study reported by Lashen et al., which showed an overall SVR‐12 of 97.8% for a population that included both naïve and treatment‐experienced patients with or without cirrhosis who received generic combinations of either Sof‐Led or Sof‐Dac.^20^ The second one compares branded and generic combinations for Sof‐Led and Sof‐Dac with an SVR‐12 in the range of 97.7%–100% regardless of treatment history degree of liver disease.^26^ The third study assessed a similar combination in both treatments' naïve and treatment‐experienced patients ± ribavirin with reported SVR‐12 in the range of 96%–100%, with lower proportions reported in patients with chronic hepatitis and cirrhosis ~89%.^20^

The CTP score in this study did not change in the majority of the patients at the time of SVR‐12, which is explained and expected because most of the patients included were classified as CTP‐A in both groups at the beginning of the study. However, treatment with DAA in one study was associated with improved CTP scores and resulted in a reversal of liver decompensation state.^27^ This would be important to decide on the urgency of treatment in patients with decompensated cirrhosis, or deferring the treatment after transplantation is a preferred approach.

Treatment of HCV recurrence postliver transplant with DAA has been reported with high SVR‐12 of ~86%–96%, and no major concerns for clinically significant drug‐drug interactions with immunosuppressants, which was similar to our study with an SVR‐12 reported between 86% and 100% in this subgroup population.^19^, ^28^, ^29^

The combination of Sof‐Led (Harvoni®) cost for 12 weeks course of therapy was around 203,868 SR per patient, while the generic sofosbuvir (Sovira®) combined with branded daclatasvir (Daclinza®) course of treatment cost was 76,524 SR, which resulted in direct cost reduction by 62%. Moreover, the cost of combining branded sofosbuvir (Sovaldi®) and branded daclatasvir (Daclinza®) for 12 weeks course of therapy was 301,140 SR, which represented a 75% potential cost inflation compared to generic sofosbuvir (Sovira®) and branded daclatasvir (Daclinza®).

This is the first prospective trial with a historical control that evaluated the effectiveness of generic sofosbuvir in combination with daclatasvir for HCV genotype 4. Our study has some limitations; first, there were differences in some baseline patients' characteristics between both groups, such as age, fibrosis score, and CTP score, which may have influenced the outcome favoring Sof‐Dac. This is mainly due to a lack of randomization. Although the study was designed as noninferiority with a 9% noninferiority margin, the differences in baseline characteristics are still of concern, making it unsuitable for making the direct comparison without acknowledging the found difference. Second, patients in group 1 received ribavirin due to higher fibrosis scores. Third, our study included a small number of postliver transplant patients, for which generalization of the results in this subpopulation cannot be made. Fourth, the sample size calculation was based on prior solid data in none transplant patients, as the data on patients treated posttransplant was scarce. Fifth, adverse event was captured in the historical group from previous documentation in the electronic patient record, which could be associated with inaccuracy. Finally, although Sof/Dac is one of the first‐line regimens in treating genotype 4 HCV according to AASLD guidelines, our results should not be extrapolated to other first‐line regimens. Because there was only one patient in the Sof‐Dac group who did not achieve an SVR‐12 response, attempting to evaluate the effect of CTP and Metavir scores on achieving SVR‐12 was not possible.

## 5 CONCLUSION

Combined with branded daclatasvir, generic sofosbuvir was safe and effective for treating genotype 4 HCV compared to Sof‐Led. This combination may significantly reduce the cost burden, enabling a larger pool of treated patients.

## AUTHOR CONTRIBUTIONS

**Hala Joharji**: Conceptualization; data curation; formal analysis; investigation; methodology; project administration. **Delal Alkortas**: Methodology; resources; validation; writing – review and editing. **Aziza Ajlan**: Data curation; resources; validation; writing – review and editing. **Mohamed Ahmed**: Data curation; validation; writing – original draft; writing – review and editing. **Hamad Al‐Ashgar**: Conceptualization; resources; supervision; writing – review and editing. **Mohammed Al‐Quaiz**: Project administration; supervision; writing – review and editing. **Dieter Broering**: Conceptualization; project administration. **Mohammed Al‐Sebayel**: Data curation; resources; validation; writing – review and editing. **Hussien Elsiesy**: Data curation; resources; writing – review and editing. **Faisal A. Alkhail**: Conceptualization; writing – review and editing. **Waleed K. Al‐Hamoudi**: Methodology; resources; Writing – review and editing. **Edward De Vol**: Conceptualization; data curation; formal analysis; resources; software; validation; writing – review and editing. **Epi Amirah Al‐Almuhayshir**: Data curation; writing – original draft. **Ahmed Al‐Jedai**: Conceptualization; formal analysis; methodology; project administration; supervision; validation; writing – original draft; writing – review and editing.

## CONFLICT OF INTEREST

The authors declare no conflict of interest.

## TRANSPARENCY STATEMENT

The lead author Ahmed Al‐Jedai affirms that this manuscript is an honest, accurate, and transparent account of the study being reported; that no important aspects of the study have been omitted; and that any discrepancies from the study as planned (and, if relevant, registered) have been explained.

## Data Availability

Data are available on request due to privacy/ethical restrictions.
